# Episodic memory and associated cortical atrophy in amnestic early-onset and late-onset Alzheimer's disease

**DOI:** 10.1177/13872877261469183

**Published:** 2026-07-23

**Authors:** Cierra M. Keith, Marc W. Haut, Patrick Worhunsky, Camila Vieira Ligo Teixeira, Rashi I. Mehta, Joseph Malone, Holly Phelps, Melanie Ward, Mark Miller, Stephanie Pockl, Nafiisah Rajabalee, Gary Marano, William T. McCuddy, Pierre-François D'Haese, Ali Rezai

**Affiliations:** 1 Rockefeller Neuroscience Institute, West Virginia University, Morgantown, WV, USA; 2 Department of Behavioral Medicine and Psychiatry, West Virginia University, Morgantown, WV, USA; 3 Department of Neurology, West Virginia University, Morgantown, WV, USA; 4 Department of Neuroradiology, West Virginia University, Morgantown, WV, USA; 5 Department of Medicine, West Virginia University, Morgantown, WV, USA; 6 Department of Radiology, West Virginia University, Morgantown, WV, USA; 7 Department of Neuropsychology, Barrow Neurological Institute, St Joseph Hospital and Medical Center, Phoenix, AZ, USA; 8 Department of Neurosurgery, West Virginia University, Morgantown, WV, USA

**Keywords:** Alzheimer's disease, cortical thickness, early-onset Alzheimer's disease, magnetic resonance imaging, memory and learning

## Abstract

**Background:**

Memory consolidation problems are often prototypical in Alzheimer's disease (AD). However, it remains undetermined whether episodic memory presents similarly in early-onset Alzheimer's disease (EOAD) relative to the more commonly occurring late-onset Alzheimer's disease (LOAD).

**Objective:**

This study examined episodic memory and its neuroanatomical correlates in amnestic early-onset (aEOAD) relative to amnestic late-onset AD (aLOAD).

**Methods:**

Using our single center data set obtained from a memory clinic setting (N = 180), we examined group differences in multiple markers of episodic memory along with associations with volume and thickness of underlying signature brain regions.

**Results:**

We did not observe any difference for examined measures of memory performance between aEOAD and aLOAD. Associations between episodic memory processes and volume and thickness of the brain regions examined were also largely similar, except for a stronger relationship between memory consolidation and thinner left supramarginal gyrus observed in the aEOAD group.

**Conclusions:**

Overall, the current results support similar memory consolidation processes in early- and late-onset amnestic Alzheimer's disease, though the parietal cortex may play a larger role in memory consolidation in aEOAD.

## Introduction

Difficulty consolidating new memories is often a prototypical symptom of Alzheimer's disease (AD). Encoding and consolidation problems are commonly associated with atrophy in the mesial temporal lobes, including the hippocampus and entorhinal cortex^1,2^; however, regions of parietal and frontal cortices have also been associated with memory in AD.^3,4^ Utilizing multiple markers of verbal episodic memory, we have previously reported that amyloid positive (Aβ +) patients with amnestic mild cognitive impairment (aMCI) demonstrate greater memory problems than their amyloid negative (Aβ -) aMCI counterparts, despite showing similar levels of hippocampal and entorhinal atrophy.^5^ Furthermore, differences in parietal cortex thickness were associated with the greater memory problems observed in Aβ+ aMCI relative to Aβ - aMCI. In this prior work, the Aβ+ individuals were not stratified by age. While the typical age of onset of clinical symptoms in AD is after 65, known as late-onset AD (LOAD), approximately 5% of patients have symptoms before the age of 65, known as early-onset AD (EOAD). EOAD can result in particularly devastating consequences with unique psychosocial and occupational challenges.^6,7^ While EOAD is less studied than LOAD, a more aggressive disease course^8^ and greater pathological burden have been documented in prior literature.^9,10^ Memory impairment often precedes decline across other cognitive domains in the classically presenting form of AD; however, a higher prevalence of nonmemory variants, including visuospatial and language predominant phenotypes, have been reported in EOAD relative to LOAD.^11,12^ Due to these atypical variants and associated non-traditional disease course with psychiatric burden, EOAD patients are oftentimes excluded from AD research, consequently making them an understudied group.

Similarities and differences in cognitive symptoms at presentation in EOAD and LOAD have been debated in existing literature. Some studies report no differences in memory performances,^8,13–16^ while others report greater memory impairment in LOAD.^17,18^ In examining recent large studies, comparison of the Longitudinal Early Alzheimer's Disease Study (LEADS) and the Alzheimer's Disease Neuroimaging Initiative (ADNI) cohorts demonstrated worse delayed recall memory composite scores in LOAD relative to EOAD.^19^ However, after removing participants with nonamnestic subtypes from the EOAD group, there were no differences observed between EOAD and LOAD for delayed recall. Data from the BioFINDER2 study^20^ noted broad similarities in atrophy and other biomarkers when comparing amnestic EOAD with amnestic LOAD, though EOAD showed thinner parietal cortex while LOAD showed thinner entorhinal cortex. These differences have also been noted by others,^21–23^ although some studies note similar atrophy patterns for EOAD and LOAD in amnestic subtypes.^24^

Using a single marker of episodic memory, delayed recall, we have previously reported no difference in memory retrieval between EOAD and LOAD, though we did observe thinner mesial temporal gyrus in LOAD. We have also replicated the EOAD cortical signature in our cohort, which is characterized by atrophy most prominently in the lateral temporal cortex, inferior parietal lobule, posterior cingulate, and precuneus, with relative sparing of the medial temporal lobe.^25^ In our prior work, we found associations between delayed recall and the average thickness of EOAD signature regions.^26^ However, we did not examine other indicators of episodic memory beyond delayed recall or relationships between memory performances and specific EOAD and LOAD signature regions. Many studies examine memory using a unitary composite measure rather than specific aspects/processes of memory.^19^ We are not aware of any previous work investigating multiple markers of memory processes in Aβ + EOAD relative to Aβ + LOAD at time of presentation to a memory clinic. Investigating memory in EOAD more comprehensively (e.g., including measures of learning, retention, discrimination, and memory errors) is crucial to better characterize possible markers of early-onset disease and identify potential differences in early- versus late-onset AD. Improving the characterization of these groups across multiple measures of memory function may have treatment implications with the advent of monoclonal antibody therapies.

In the current study, we sought to examine episodic memory processes in depth in amnestic EOAD (aEOAD) relative to amnestic LOAD (aLOAD) using our large, well-characterized, single center data obtained from our multidisciplinary memory clinic. To maintain the focus on the LOAD/EOAD comparison, we did not include control participants, as differences in memory performances with controls is well-documented. In addition, we only included patients with evidence of memory impairment on cognitive evaluation rather than pure nonamnestic subtypes. Memory is a complex process that entails encoding, storage, and retrieval of information, and involves multifocal brain regions including the medial temporal lobe and neocortex.^27^ As such, we examined multiple markers of memory using the California Verbal Learning Test – II short form: initial learning level, delayed recall, percentage of information retained over a delay, number of intrusion errors, number of false positives on recognition, and ability to discriminate positive from negative items on recognition. Many studies have relied on delayed recall as a classic measure of memory consolidation over time; however, examining learning across trials provides important context with which to consider delayed recall. We therefore opted to incorporate percentage retained after the delay. To determine if the information was stored but insufficiently retrieved, we used recognition discrimination. Impaired recognition discrimination in the face of poor delayed recall and/or low percentage of information retained supports impaired memory consolidation. We also examined recall intrusion and false positive memory errors, which have been associated with incomplete or faulty consolidation of information.^28,29^ We have previously shown associations between these measures and volume/thickness of multiple regions including hippocampus, and superior frontal gyrus.^4,5^

We then examined the neuroanatomical correlates underlying episodic memory using cortical thickness of previously identified signature cortical regions^25,26,30^ associated with memory. We included the volume of the hippocampus, along with cortical thickness of the following regions: entorhinal cortex, middle temporal gyrus, superior frontal cortex, inferior parietal, supramarginal gyrus, and precuneus. We hypothesized that while comparable delayed recall has been observed in LEADS and ADNI cohorts,^19^ differences in aEOAD and aLOAD may emerge in examining additional measures of the memory process. We also predicted that consistent with prior studies, aLOAD would demonstrate reduced volume or thickness of the hippocampus, entorhinal cortex, and middle temporal gyrus, while aEOAD would demonstrate thinner cortex in parietal regions. We further examined associations between memory performances, neuroanatomical variables, and diagnostic group to identify any main effects or interactions. We hypothesized that volume or thickness in the differing regions between aEOAD and aLOAD would also differentially relate to episodic memory performances (i.e., temporal lobe associations in aLOAD, and parietal lobe associations in aEOAD). The examination of multiple memory variables in a large, well-characterized memory clinic cohort of aEOAD and aLOAD is a novel contribution to existing literature.

## Methods

### Participants

Participants (N = 180, 111 = aLOAD and 69 = aEOAD) were comprised of a naturalistic sample of patients with aMCI or dementia due to underlying AD^31^ seen in the Memory Health Clinic at the Rockefeller Neuroscience Institute of West Virginia University from June 2020 through May 2025. Patients were diagnosed by consensus of a team comprised of neuropsychologists, neurologists, psychiatrists, geriatricians, and neuroradiologists. Inclusion criteria were i) evidence of amnestic-domain impairment (1 or more standard deviations below normative performance on at least one memory measure), ii) a high-resolution MRI of sufficient quality for morphometric analysis, and iii) amyloid positive status by ^18^F-florbetaben PET. Patients with multiple primary etiologies were excluded.

Participants aged 66 years or younger (N = 69) at the time of their initial clinic visit were classified as aEOAD. The rationale for this age is that patients who present to the clinic typically report at least one year of symptoms when considering recognition of symptoms by patient and/or family and wait time to been seen in the clinic. Participants provided written informed consent for the use of their clinical data for research approved by the West Virginia University IRB.

### Memory performance

The short form of the California Verbal Learning Test, 2nd edition (CVLT-II-SF^32^) was used to examine multiple measures of the memory process. This test involves learning a list of nine words from three semantic categories over four learning trials. After recall of the fourth trial, there is free recall trial following a 30-s distraction task and then a long delay free recall trial after 10 min. Cued recall is conducted using semantic cues followed by a yes/no recognition trial.

CVLT initial learning measures of interest included the total number of words recalled (Total) and a learning score that accounts for the maximum number of words that could be learned relative to baseline performance (Learning).^33,34^ Performance measures following the 10-min delay period included the number of words correctly recalled in total (Delay Recall) and as a percentage of words recalled during the fourth learning trial (Retention). Performance during the yes/no recognition trial was measured using the discriminability index based on both recognition hits and false positives (Discriminability) and the total number of false positives separately (False Positives). The number of intrusion responses were summed across all learning and recall trials (Intrusions).

### Additional clinical characteristics

*APOE* genotyping data were available for 176 patients. Additional measures examined to characterize the sample included: i) the Mini-Mental State Examination (MMSE; N = 180),^35^ a measure of overall cognitive status and degree of impairment; ii) the Word Reading subtest of the Wide Range Achievement Test, 4th Edition (WRAT-4; N = 173),^36^ an estimate of premorbid intellectual functioning; iii) the Functional Activities Questionnaire (FAQ; N = 151),^37^ an assessment of functional impairment in patient populations; and iv) the Neuropsychiatric Inventory Questionnaire (NPI-Q; N = 123),^38^ a measure of neuropsychiatric symptoms. All participants completed the MMSE as part of their routine clinical examination. Other measures were examined for varying subsets of participants completing each assessment. Multidomain symptoms (versus single domain amnestic) were characterized based on neuropsychological assessment results.

### MRI acquisition and processing

T1w and T2w FLAIR MRI images were acquired on either a Siemens 3 T Magnetom Prisma scanner with a 20-channel head coil (aLOAD N = 106, aEOAD N = 65; e.g., T1w MPRAGE;TR/TE = 2300/2.26 ms, TI = 900 ms, flip angle=8, acceleration factor=2) or a GE Architect 3 T scanner with a 48-channel coil (aLOAD N = 5, aEOAD N = 4; e.g., T1w Bravo SPGR; TR/TE = 8.5/3.3 ms, TI = 45450 ms, flip angle=12, acceleration factor=2). Scanner type was equally distributed across groups (χ^2^ = 0.15, p = 0.70). T1w images were collected at a 1mm-isotropic (aLOAD N = 25, aEOAD N = 15) or sub-millimeter in-plane (aLOAD N = 86, aEOAD N = 54) resolution which was equally distributed across groups (χ^2^ = 0.02, p = 0.90).

Brain MRI measures of interest from T1w images (i.e., hippocampal volume and cortical thickness) were measured using FreeSurfer v7.4.10 (http://surfer.nmr.mgh.harvard.edu) following the methods described in our previous work.^26^ Briefly, images were inspected for motion and/or signal artifact prior to undergoing intensity normalization, skull stripping, and automated segmentation for demarcation of the grey and white matter boundaries and pial surfaces. Segmentations were visually inspected for errors and manual corrections were performed as needed. Cortical thickness was calculated as the closest distance from the grey/white boundary to the grey/cerebrospinal fluid (CSF) boundary at each surface vertex. Thickness and volume estimates across the whole brain were harmonized with respect to imaging sequence (i.e., resolution and orientation) using ComBat (github.com/rpomponio/neuroHarmonize).^39^

The Desikan-Killiany atlas^40^ was used to identify regions of interest (ROIs) including the middle temporal, precuneus, inferior parietal, and supramarginal gyri. We also included the superior frontal gyri, as it has been reported to interact with medial temporal structures for aspects of memory. We additionally included entorhinal thickness as well as hippocampal volume given the well-established relationships between these regions and memory processes in AD. Hippocampal volumes were assessed as a percentage of the total intracranial volume.^41^ Regional thickness and volume were examined by hemisphere. The total volume of white matter hyperintensities was measured from T2w FLAIR images using the lesion segmentation toolbox.^42^

### Amyloid PET acquisition and processing

Amyloid PET was acquired with a Siemens Biograph mCT system 90 min post-injection of 8.0 mCI of ^18^F-florbetaben. Images were reconstructed with an ordered subset-expectation maximization algorithm embedded in the Siemens workstations. PET images were co-registered with T1w MRI images using CT scans acquired at the time of the PET. Quantification of amyloid levels was performed with regional segmentation of the T1w image using SLANT.^43^ Average whole-brain cortical SUVr was computed with reference to average SUV in cerebellar gray matter. For study inclusion, Aβ positivity was defined as an average whole-brain cortical SUVr ≥ 1.18 for aEOAD and SUVr ≥ 1.20 for aLOAD.^25,44,45^

### Statistical analysis

Group differences in demographic characteristics, standard clinical measures, and CVLT-II performances were examined using Chi-square or t-tests as appropriate. Differences in MRI T1w brain measures (volume or thickness of regions described above) were assessed in univariate analyses adjusting for sex and age. The relationship between diagnostic group in the associations between memory performance and MRI brain measures were investigated using MANCOVA analyses. The seven CVLT-II outcome measures were individually Z-scaled and entered together into separate models for each MRI brain measure of interest. All models included factors of diagnostic group and sex, and population-centered covariates for the MRI brain measure, age, amyloid SUVr, and white matter hyperintensity volumes. Group interaction terms were included for all predictors. Any overall effects of interest (i.e., effect of MRI-measure and group-by-MRI-measure interaction on CVLT-II scores), were followed up with post-hoc testing as appropriate. Correction for multiple comparisons across the fourteen brain measures (seven regions, right and left hemisphere) were performed using a false-discovery rate at p < 0.05 with uncorrected results reported as exploratory findings.

Bayesian multivariate regression models were conducted for each ROI to quantify evidence for the absence of ROI effects using the brms package^46^ in R (v4.5.3, R Core Team, 2025). Evidence regarding the overall ROI block was examined by comparing the adjusted base model alone with a model adding the ROI main effect and Group*ROI interaction. Evidence regarding the Group*ROI interaction specifically was examined by comparing the adjusted ROI main-effect model with a model including the interaction. BF01 values over 10 were interpreted as strong evidence favoring the simpler model.

## Results

Demographic and clinical characteristics, and neurocognitive performances for the 180 participants (aEOAD N = 69, aLOAD N = 111) are provided in Table 1. Briefly, aEOAD and aLOAD participants were similar on demographic and clinical measures (e.g., MMSE, FAQ, NPI-Q), with the exception of age (which was used to differentiate patients into groups; t = 19.23, p < 0.001), and white-matter hyperintensity volumes (t = 3.70, p < 0.001). Notably, without age adjustments, groups did not differ in memory performance, general neurocognitive functioning, or whole-brain cortical amyloid SUVr (p-values>0.20). Adjusting for sex and age, there were no differences between groups in ROI volumes or cortical thickness (p-values>0.15; Supplemental Table 1).

**Table 1.** Participant characteristics and memory performance.

	aEOAD	aLOAD	t /χ^2^
Age, years (SD)	59.3 (5.1)	72.4 (4.0)	19.23**
Female, N (%)	37 (53.6)	60 (54.1)	0.00
Education, years (SD)	14.6 (2.5)	14.8 (2.7)	0.62
*Clinical characteristics*			
Dementia, N (%)	10 (14.5)	27 (24.3)	2.52
*APOE4* carrier^a^, N (%)	32 (47.1)	62 (57.4)	1.80
Multi-domain symptoms, N (%)	57 (82.6)	94 (84.7)	0.14
MMSE, total (SD)	26.5 (3.0)	27.0 (2.3)	1.33
WRAT, reading (SD)	100.4 (13.5)	102.4 (13.1)	0.95
FAQ^b^, total (SD)	6.7 (7.1)	6.6 (5.5)	0.13
NPI-Q^c^, total (SD)	3.8 (2.5)	3.5 (2.6)	0.67
*Clinical imaging measures*			
Sub-millimeter T1w, N (%)	54 (78.3)	86 (77.5)	0.02
WM hyperintensity, mL (SD)	2.0 (2.8)	4.3 (4.7)	3.70**
Whole-brain amyloid, SUVr (SD)	1.5 (0.4)	1.6 (0.3)	1.58
*CVLT performance, M (SD)*			
Total	23.1 (6.1)	22.6 (5.1)	0.64
Learning	13.4 (7.4)	12.8 (7.2)	0.53
Delay Recall	4.5 (2.9)	4.0 (2.7)	1.22
Retention	0.6 (0.4)	0.6 (0.4)	1.03
Discriminability	2.2 (0.9)	2.2 (0.9)	0.16
False Positives	2.5 (2.6)	2.6 (2.6)	0.35
Intrusions	3.0 (3.1)	3.2 (3.3)	0.39

MMSE: Mini-Mental State Examination; WRAT-4: Wide Range Achievement Test, 4th Edition (reading subscale score); FAQ: Functional Activities Questionnaire; NPI-Q: Neuropsychiatric Inventory Questionnaire; WM: white matter; CVLT: California Verbal Learning Test, 2nd edition (short form). Assessment citations and descriptions of CVLT performance measures are provided in text. Data available on a subset of participants (aEOAD N, aLOAD N): ^a^(68,108); ^b^(52,99); ^c^(41,82).

**p < 0.01, *p < 0.05.

**Figure 1.**
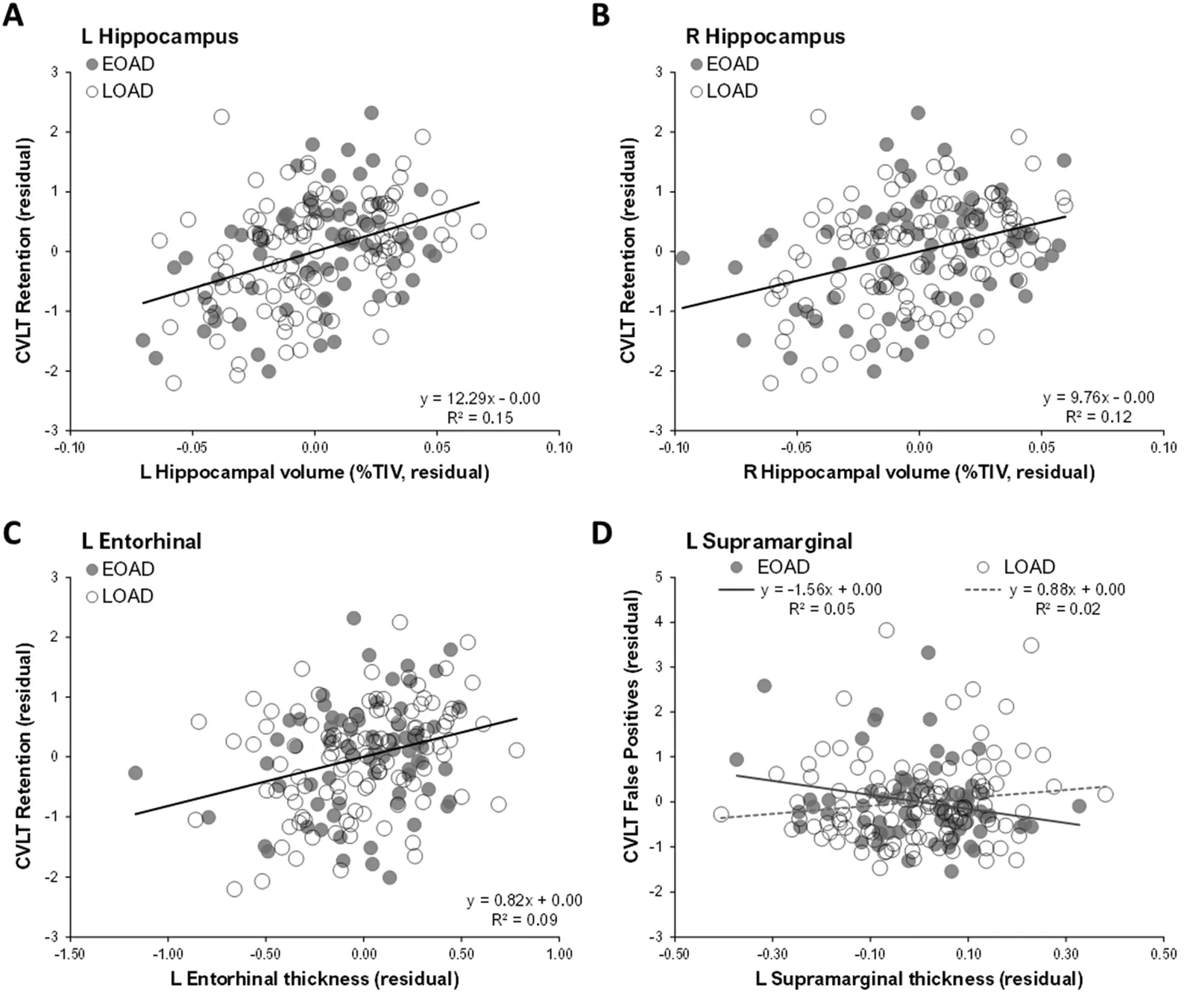
The associations between hippocampal volume and a representative CVLT-II measure (Retention) are shown in panel A and B. The association between left entorhinal thickness and a representative CVLT-II measure (Retention) is shown in panel C. The significant group × region interaction involving false positive errors and the left supramarginal gyrus is shown in panel D.

Positive results of MANCOVA analyses investigating associations between group, memory performance, and MRI brain measures of interest are presented in Table 2 with full statistics across all 14 regions. There were associations between memory performance and hippocampal volumes bilaterally (left: F[7162] = 7.55, p < 0.001; right: F[7162] = 6.64, p < 0.001) that did not interact with group (left: F[7162] = 1.52, p = 0.16; right: F[7162] = 0.92, p = 0.49). Post-hoc analyses revealed smaller hippocampal volumes were associated with lower Delay Recall (left: F[1168] = 34.50, p < 0.001; right: F[1168] = 27.61, p < 0.001), Retention (left: F[1168] = 29.69, p < 0.001; right: F[1168] = 22.25, p < 0.001) and Discriminability (left: F[1168] = 30.80, p < 0.001; right: F[1168] = 25.46, p < 0.001), and more False Positives (left: F[1168] = 34.36, p < 0.001; right: F[1168] = 25.07, p < 0.001) and Intrusions (left: F[1168] = 23.32, p < 0.001; right: F[1168] = 21.93, p < 0.001). The association between hippocampal volume and a representative CVLT-II measure (Retention) are shown in Figure 1A and 1B. Associations between smaller hippocampal volumes and lower Learning scores (left: F[1168] = 7.03, p = 0.009; right: F[1168] = 5.73, p = 0.018) did not survive FDR correction.

**Table 2.** Primary MANCOVA results and Bayesian evidence for ROI block and Group*ROI interaction effects.

	Region		Group × region		BF01	
Region	F	p	F	p	ROI Block	Group*ROI
L Hippocampus	7.55	<0.001	1.52	0.163	0.4	0.6
R Hippocampus	6.64	<0.001	0.92	0.491	0.3	0.8
L Entorhinal	3.05	0.005	1.38	0.216	106	43
R Entorhinal	1.58	0.146	0.33	0.938	609342	1473
L Inferior parietal	1.12	0.355	1.38	0.218	569	5.7
R Inferior parietal	1.16	0.331	1.12	0.353	369	6.5
L Middle temporal	1.99	0.059	0.63	0.734	319	24
R Middle temporal	0.55	0.795	0.43	0.883	5924	29
L Precuneus	0.60	0.758	1.04	0.406	2867	9.7
R Precuneus	0.35	0.932	0.84	0.553	3083	9.6
L Superior frontal	0.39	0.908	1.04	0.405	2149	6.0
R Superior frontal	0.66	0.707	1.51	0.167	185	1.8
L Supramarginal	1.74	0.103	2.11	0.046	8.7	0.9
R Supramarginal	0.63	0.727	0.40	0.899	3000	23

BF01 values quantify evidence favoring the simpler model in each Bayesian comparison, with “ROI block” comparing the base model alone with a model adding the ROI main effect and Group × ROI interaction and “Group × ROI” comparing models with and without the interaction term specifically. BF01 > 10 was interpreted as strong evidence supporting the simpler model.

There was a similar association between memory performance and left entorhinal cortex thickness (F[7162] = 3.05, p = 0.005) that did not interact with group (F[7162] = 1.38, p = 0.21). Post-hoc analyses revealed thinner left entorhinal cortex was associated with lower Delay Recall (F[1168] = 11.89, p < 0.001), Retention (F[1168] = 13.74, p < 0.001) and Discriminability (F[1168] = 9.71, p = 0.002, and more False Positives (F[1168] = 13.43, p < 0.001) and Intrusions (F[1168] = 11.82, p < 0.001). The association between left entorhinal thickness and a representative CVLT-II measure (Retention) is shown in Figure 1C.

There was a group-by-region interaction on memory performance involving the thickness of the left supramarginal gyrus (F[7162] = 2.11, p = 0.046) that did not survive FDR correction. Post-hoc analyses indicated the interaction was significant for False Positives (F[1168] = 5.13, p < 0.024), with tendencies for thinner supramarginal gyri in to be associated with more False Positives in aEOAD (β=-1.56, se = 0.84; F[1,63] = 3.48, p = 0.067) (Figure 1D).

Bayesian comparison BF01 values for the overall ROI block favored the base model for most cortical ROIs, but not for bilateral hippocampus or left supramarginal gyrus (Table 2, Supplemental Table 9). In the interaction-specific comparison, BF01 values provided strong evidence favoring the simpler no-interaction model for left entorhinal, right entorhinal, left middle temporal, right middle temporal, and right supramarginal ROIs (Table 2, Supplemental Table 10). Evidence favoring the no-interaction model was weaker for inferior parietal, superior frontal, hippocampal, and left supramarginal ROIs.

## Discussion

In the current study, we aimed to examine whether there are significant differences in verbal episodic memory problems in amnestic EOAD relative to LOAD. We hypothesized that memory performances would be worse in aLOAD, and that differences in volume or thickness of certain brain regions would explain these differences. In contrast, we did not observe significant differences between aEOAD and aLOAD on any examined measures of the memory process. In addition, we did not observe differences between the groups for hippocampal volume or cortical thickness of any examined signature areas. However, consistent with our hypothesis, there was a significant interaction between group (aEOAD versus aLOAD) and MRI brain region, such that consolidation was differentially associated with the thickness of the left supramarginal gyrus. Specifically, thinner left supramarginal gyrus in aEOAD tended to associate with more false positive errors, though this effect was not observed for other parietal regions (i.e., inferior parietal or precuneus). In addition, there were no group by region interactions involving the hypothesized temporal regions in aLOAD. Thus, our findings provide some, albeit limited, support for differences in the neuroanatomical underpinnings of memory in aEOAD relative to aLOAD. However, the majority of our findings were suggestive of more similarities between these groups regarding memory severity and neuroanatomical associations. Bayesian model comparisons provided additional context for the largely nonsignificant Group*ROI interactions. Interaction-specific BF01 values supported the simpler no-interaction model for several cortical regions, although evidence was not uniformly strong across all ROIs. These results lend further support that many ROI-memory associations are broadly similar across aEOAD and aLOAD, while also indicating that the present data do not provide equally strong Bayesian evidence for equivalence across all regions.

Results of previous studies examining memory function in EOAD and LOAD are mixed,^8,13,17,19^ and most studies have utilized delayed recall performance only, rather than more comprehensive measures of the memory process. The present study is the first to our knowledge to compare memory performances with the inclusion of learning, retention, discriminability, and memory errors on a list learning task in aEOAD relative to aLOAD. Amnestic EOAD did not differ from LOAD in any of these various components of the memory process (i.e., learning, retention, discriminability, false positive, or intrusion errors). These data expand upon our prior work showing no difference in delayed recall performance when comparing EOAD with LOAD,^26^ and other previous studies that also demonstrated similar memory performances in early- and late-onset Alzheimer's disease.^8,13,15,16^ It is important to note that aEOAD and aLOAD groups did not differ in memory performance, general neurocognitive functioning, or whole-brain cortical amyloid SUVr even without age adjustments. This is relevant to interpretation in that similar raw score memory performances in aEOAD and aLOAD may appear more striking in the early-onset group, particularly when compared with age-matched peers. It is also important to note that our EOAD sample was comprised of only patients with memory impairment (aEOAD), thereby reducing potential biases related to atypical presentations driving differences in memory performance or atrophy patterns. Results may differ in a sample of predominantly nonamnestic EOAD. The included aEOAD sample was also mildly affected with the majority having aMCI; this is crucial to investigate given the increasing value of early diagnosis in the era of new disease modifying therapies.

Expected differences in volume and thickness of signature areas were not observed between aLOAD and aEOAD for any of the examined brain regions. While reduced medial temporal volumes in LOAD relative to EOAD have been previously reported,^23^ broadly similar structural volume loss between these groups has also been supported by multiple prior studies.^26,47^ The present findings add to the body of literature suggesting similar neuroanatomical structural impacts regardless of age of AD onset. Differences in medial temporal structures, such as the hippocampus, may emerge with the inclusion of nonamnestic variants within the EOAD group with potential increased likelihood of hippocampal sparing.^48^ The disproportionate presence of atypical variants in EOAD adds complexity to its study and interpretation, as many prior studies include both amnestic and nonamnestic variants. Regarding relationships between the volume and thickness of brain regions of interest and memory performances, expected associations were observed for measures of memory and temporal lobe regions, including the hippocampus and entorhinal cortex. Significant associations were primarily observed for left-sided temporal regions (though associations with the hippocampus were bilateral), which is unsurprising given the verbal nature of the examined task. Although many studies have observed relationships between memory and brain regions outside of the temporal lobes,^4,49^ when focusing solely on classically presenting aEOAD and aLOAD, the present study suggests that primary effects are observed for the hippocampus and entorhinal cortex.

When examining interaction effects, a single significant group by region effect was observed for the supramarginal gyrus. Post hoc analyses revealed that more false positive errors were related to thinner supramarginal gyrus in aEOAD. This finding must be interpreted with caution, as it did not survive correction, but does lend partial support to our hypothesis that the parietal lobe may relate more strongly to memory in aEOAD relative to aLOAD. There is a considerably large literature base supporting the importance of the parietal lobe in memory functions broadly^50–52^ and in AD.^53,54^ There is also an existing literature base indicating greater parietal lobe involvement specifically in EOAD; some posit that increased amyloid burden and metabolic dysfunction in the parietal lobe in EOAD may account for differences in presentation and course relative to LOAD.^55^ The cortical signature for EOAD also contains a notable predominance of parietal and posterior temporal regions.^25,26^ Further research is needed to clarify the specific role of the parietal lobe in memory processes both in LOAD and EOAD as well as how this relationship might change with differing clinical phenotypes and disease progression.

Overall, in the setting of mixed findings in prior studies comparing EOAD with LOAD, the current findings add support to similar memory impairments and shared neuroanatomical underpinnings when isolating amnestic subtypes for LOAD and EOAD. These results align with two recent studies reporting comparable delayed memory performance^19^ and similar atrophy patterns^20^ when only examining the amnestic subtype of EOAD relative to LOAD. Clinically, our groups were well-matched for MMSE, FAQ, *APOE4*, estimated premorbid functioning, and sex. Additionally, we controlled for amyloid level and white matter hyperintensities to reduce these potential confounds. It is important to note differences in our sample relative to those in prior studies examining memory and neuroimaging findings in EOAD and LOAD. For instance, our aEOAD sample is notably earlier in the disease process than the LEADS^19^ consortium EOAD sample, which may suggest that differences in cognition and neuroanatomical underpinnings may become more apparent with disease progression. Cognitive differences may also be more apparent in nonamnestic domains, as is supported by prior research showing similar trajectory of memory decline in EOAD and LOAD with faster rate of decline in EOAD for nonamnestic domains.^8^ Taken together, the present findings emphasize the importance of considering clinical phenotype and disease stage in addition to age of onset; such considerations may have implications regarding clinical endpoints for aEOAD and aLOAD. Further work is warranted to more directly identify differences in diagnostic and treatment outcomes in these groups.

This study is not without limitations. First, there are methodological considerations that must be acknowledged when using a real-word clinical sample (e.g., degree of independence between dependent and independent variables, biases in clinical decision making, etc.). Nonetheless, we have aligned our methodology and group characterization with other large consortium studies^19^ in our wholistic utilization of clinical symptoms, biomarkers, cognitive data, and multidisciplinary consensus review for diagnosis. Such real-world samples remain crucial for tailoring clinical implications. Second, we did not have access to tau biomarker data. It is possible that tau levels could account for group differences that measures of atrophy/neurodegeneration did not reflect. Indeed, there are studies that show greater tau is associated with EOAD.^56^ Included participants were also not tested for dominantly inherited genetic mutations such as APP and PSEN. Next, this is a cross-sectional study, and longitudinal analyses will be necessary to delineate the trajectory of memory performance and underlying atrophy over time. In addition, our sample is not racially diverse but does represent a rural Appalachian sample which is not well studied. Replication with more diverse samples is recommended. We used a verbal memory test and as such, our effects were primarily left hemisphere-based and may not generalize to different modalities of memory stimuli. Finally, we did not include comparison groups of young and older participants with memory impairment but without amyloid biomarker positivity, as we chose to focus exclusively on age-based differences in memory performances in amnestic AD.

In conclusion, the results of this study suggest that among individuals with aEOAD and aLOAD who present with memory impairment, performances across multiple markers of episodic memory do not significantly differ between groups. There was limited evidence that age of onset moderates the relationship between volume/thickness of signature regions and memory, with Bayesian model comparisons providing region-specific support for the absence of Group*ROI interactions. Consistent with prior findings, memory in aEOAD may be more associated with parietal atrophy than in aLOAD, though this finding requires additional investigation. With this exception, memory performances and their neuroanatomical underpinnings may be largely similar for EOAD and LOAD when memory impairment is present. Longitudinal investigations using large, well-characterized EOAD cohorts are needed to further our understanding of clinical presentation across phenotypic variants and how they differ from LOAD, with the goal of promoting earlier diagnosis and treatment access.

## Supplemental Material

sj-docx-1-alz-10.1177_13872877261469183 - Supplemental material for Episodic memory and associated cortical atrophy in amnestic early-onset and late-onset Alzheimer's disease Supplemental material, sj-docx-1-alz-10.1177_13872877261469183 for Episodic memory and associated cortical atrophy in amnestic early-onset and late-onset Alzheimer's disease by Cierra M. Keith, Marc W. Haut, Patrick Worhunsky, Camila Vieira Ligo Teixeira, Rashi I. Mehta, Joseph Malone, Holly Phelps, Melanie Ward, Mark Miller, Stephanie Pockl, Nafiisah Rajabalee, Gary Marano, William T. McCuddy, Pierre-François D'Haese and Ali Rezai in Journal of Alzheimer's Disease
