# Supplementary material for: Episodic memory and associated cortical atrophy in amnestic early-onset and late-onset Alzheimer's disease
Source: J Alzheimers Dis. 2026 Jul 23;113(1):403–13. doi: 10.1177/13872877261469183 (PMC13499921; doi:10.1177/13872877261469183)
Supplement: Supplementary file 1 [file sj-docx-1-alz-10.1177_13872877261469183.docx]

**Supplemental Material**

**Episodic memory and associated cortical atrophy in amnestic early-onset and late-onset Alzheimer’s disease**

**Supplemental Table 1**. Mean (SD) ROI volume and cortical thickness

| **Region** | **EOAD** | **LOAD** | **F** | **η²p** |
| --- | --- | --- | --- | --- |
| L Hippocampus^a^ | 0.26 (0.03) | 0.25 (0.03) | 0.07 | 0.000 |
| L Entorhinal | 3.03 (0.32) | 2.94 (0.36) | 0.00 | 0.000 |
| L Inferior parietal | 2.23 (0.15) | 2.21 (0.13) | 1.50 | 0.008 |
| L Middle temporal | 2.55 (0.16) | 2.49 (0.14) | 1.82 | 0.010 |
| L Precuneus | 2.22 (0.17) | 2.19 (0.13) | 0.15 | 0.001 |
| L Superior frontal | 2.44 (0.13) | 2.42 (0.14) | 0.08 | 0.000 |
| L Supramarginal | 2.32 (0.15) | 2.27 (0.15) | 0.20 | 0.001 |
| R Hippocampus^a^ | 0.27 (0.04) | 0.26 (0.04) | 0.30 | 0.002 |
| R Entorhinal | 3.12 (0.35) | 3.03 (0.37) | 1.70 | 0.010 |
| R Inferior parietal | 2.28 (0.18) | 2.26 (0.14) | 0.17 | 0.001 |
| R Middle temporal | 2.62 (0.17) | 2.56 (0.14) | 0.16 | 0.001 |
| R Precuneus | 2.24 (0.16) | 2.22 (0.12) | 0.03 | 0.000 |
| R Superior frontal | 2.37 (0.11) | 2.38 (0.14) | 0.03 | 0.000 |
| R Supramarginal | 2.38 (0.14) | 2.33 (0.15) | 0.00 | 0.000 |

^a^ volume as percent TIV

**Supplemental Table 2.** Univariate CVLT results: Hippocampal volume

|  | **Left Hippocampus** | | | | | **Right Hippocampus** | | | | | |  |
| --- | --- | --- | --- | --- | --- | --- | --- | --- | --- | --- | --- | --- |
| **CVLT / Effect** | **B (SE)** | **95% CI** | **F** | **p** |  | | **B (SE)** | **95% CI** | **F** | **p** |  | |
| Total |  |  |  |  |  | |  |  |  |  |  | |
| Region | 2.95 (3.13) | -3.22, 9.12 | 5.35 | 0.022 | * | | 3.09 (2.95) | -2.74, 8.92 | 2.98 | 0.086 |  | |
| Group * Region | 5.95 (5.12) | -4.16, 16.05 | 1.35 | 0.247 |  | | 1.57 (4.49) | -7.29, 10.43 | 0.12 | 0.727 |  | |
| Learning |  |  |  |  |  | |  |  |  |  |  | |
| Region | 2.22 (3.19) | -4.08, 8.53 | 7.03 | 0.009 | ** | | 3.43 (3.01) | -2.52, 9.38 | 5.73 | 0.018 | * | |
| Group * Region | 9.43 (5.23) | -0.90, 19.76 | 3.25 | 0.073 |  | | 4.10 (4.58) | -4.94, 13.14 | 0.80 | 0.372 |  | |
| Delay Recall |  |  |  |  |  | |  |  |  |  |  | |
| Region | 31.12 (7.48) | 16.36, 45.88 | 34.50 | 0.000 | *** | | 30.18 (7.12) | 16.12, 44.23 | 27.61 | 0.000 | *** | |
| Group * Region | 9.72 (12.25) | -14.47, 33.90 | 0.63 | 0.429 |  | | -3.50 (10.82) | -24.86, 17.86 | 0.11 | 0.747 |  | |
| Retention |  |  |  |  |  | |  |  |  |  |  | |
| Region | 11.62 (2.81) | 6.08, 17.16 | 29.69 | 0.000 | *** | | 10.83 (2.68) | 5.55, 16.12 | 22.25 | 0.000 | *** | |
| Group * Region | 1.81 (4.60) | -7.27, 10.88 | 0.15 | 0.695 |  | | -2.48 (4.07) | -10.51, 5.55 | 0.37 | 0.543 |  | |
| Discriminability |  |  |  |  |  | |  |  |  |  |  | |
| Region | 14.17 (2.78) | 8.69, 19.65 | 30.80 | 0.000 | *** | | 13.07 (2.64) | 7.87, 18.28 | 25.46 | 0.000 | *** | |
| Group * Region | -3.10 (4.55) | -12.08, 5.88 | 0.46 | 0.497 |  | | -5.92 (4.01) | -13.83, 1.99 | 2.19 | 0.141 |  | |
| False Positives |  |  |  |  |  | |  |  |  |  |  | |
| Region | -11.57 (2.87) | -17.23, -5.91 | 34.36 | 0.000 | *** | | -10.86 (2.75) | -16.28, -5.43 | 25.07 | 0.000 | *** | |
| Group * Region | -4.40 (4.70) | -13.68, 4.87 | 0.88 | 0.350 |  | | 0.80 (4.18) | -7.45, 9.05 | 0.04 | 0.848 |  | |
| Intrusions |  |  |  |  |  | |  |  |  |  |  | |
| Region | -13.03 (3.08) | -19.10, -6.96 | 23.32 | 0.000 | *** | | -11.64 (2.90) | -17.36, -5.91 | 21.93 | 0.000 | *** | |
| Group * Region | 1.72 (5.04) | -8.23, 11.67 | 0.12 | 0.733 |  | | 2.62 (4.41) | -6.09, 11.33 | 0.35 | 0.553 |  | |

**p<0.01, *p<0.05

**Supplemental Table 3.** Univariate CVLT results: Entorhinal thickness

|  | **Left Entorhinal** | | | | | **Right Entorhinal** | | | | | |  |
| --- | --- | --- | --- | --- | --- | --- | --- | --- | --- | --- | --- | --- |
| **CVLT / Effect** | **B (SE)** | **95% CI** | **F** | **p** |  | | **B (SE)** | **95% CI** | **F** | **p** |  | |
| Total |  |  |  |  |  | |  |  |  |  |  | |
| Region | -0.04 (0.27) | -0.57, 0.50 | 2.34 | 0.128 |  | | 0.18 (0.26) | -0.32, 0.69 | 0.55 | 0.461 |  | |
| Group * Region | 0.78 (0.46) | -0.13, 1.68 | 2.87 | 0.092 |  | | -0.05 (0.43) | -0.90, 0.79 | 0.01 | 0.906 |  | |
| Learning |  |  |  |  |  | |  |  |  |  |  | |
| Region | -0.04 (0.28) | -0.59, 0.51 | 1.70 | 0.195 |  | | 0.47 (0.26) | -0.05, 0.99 | 2.59 | 0.110 |  | |
| Group * Region | 0.69 (0.47) | -0.24, 1.63 | 2.16 | 0.144 |  | | -0.23 (0.44) | -1.09, 0.63 | 0.28 | 0.596 |  | |
| Delay Recall |  |  |  |  |  | |  |  |  |  |  | |
| Region | 1.82 (0.68) | 0.47, 3.17 | 11.89 | 0.001 | ** | | 1.66 (0.65) | 0.37, 2.95 | 3.91 | 0.050 |  | |
| Group * Region | 0.34 (1.16) | -1.94, 2.62 | 0.09 | 0.767 |  | | -1.18 (1.09) | -3.32, 0.97 | 1.18 | 0.280 |  | |
| Retention |  |  |  |  |  | |  |  |  |  |  | |
| Region | 0.89 (0.25) | 0.39, 1.38 | 13.74 | 0.000 | *** | | 0.66 (0.24) | 0.18, 1.14 | 3.98 | 0.048 | * | |
| Group * Region | -0.20 (0.42) | -1.04, 0.63 | 0.23 | 0.633 |  | | -0.52 (0.40) | -1.31, 0.28 | 1.67 | 0.199 |  | |
| Discriminability |  |  |  |  |  | |  |  |  |  |  | |
| Region | 0.75 (0.25) | 0.25, 1.26 | 9.71 | 0.002 | ** | | 0.59 (0.24) | 0.10, 1.07 | 3.64 | 0.058 |  | |
| Group * Region | -0.17 (0.43) | -1.02, 0.68 | 0.15 | 0.701 |  | | -0.40 (0.41) | -1.20, 0.40 | 0.97 | 0.327 |  | |
| False Positives |  |  |  |  |  | |  |  |  |  |  | |
| Region | -0.62 (0.26) | -1.13, -0.10 | 13.43 | 0.000 | *** | | -0.55 (0.25) | -1.05, -0.06 | 3.52 | 0.062 |  | |
| Group * Region | -0.39 (0.44) | -1.26, 0.49 | 0.76 | 0.385 |  | | 0.32 (0.42) | -0.50, 1.15 | 0.59 | 0.443 |  | |
| Intrusions |  |  |  |  |  | |  |  |  |  |  | |
| Region | -0.95 (0.27) | -1.49, -0.41 | 11.82 | 0.001 | ** | | -0.74 (0.26) | -1.25, -0.22 | 7.92 | 0.005 | ** | |
| Group * Region | 0.31 (0.46) | -0.60, 1.22 | 0.44 | 0.506 |  | | 0.26 (0.43) | -0.60, 1.11 | 0.35 | 0.556 |  | |

**p<0.01, *p<0.05

**Supplemental Table 4.** Univariate CVLT results: Inferior parietal thickness

|  | **Left Inferior parietal** | | | | | | **Right Inferior parietal** | | | | | |
| --- | --- | --- | --- | --- | --- | --- | --- | --- | --- | --- | --- | --- |
| **CVLT / Effect** | **B (SE)** | **95% CI** | **F** | **p** |  | **B (SE)** | | **95% CI** | **F** | **p** |  |  |
| Total |  |  |  |  |  |  | |  |  |  |  |  |
| Region | 0.44 (0.72) | -0.98, 1.86 | 4.07 | 0.045 | * | 0.76 (0.66) | | -0.55, 2.07 | 4.06 | 0.045 | * |  |
| Group * Region | 1.38 (1.12) | -0.82, 3.58 | 1.53 | 0.218 |  | 0.58 (1.05) | | -1.48, 2.65 | 0.31 | 0.577 |  |  |
| Learning |  |  |  |  |  |  | |  |  |  |  |  |
| Region | 0.15 (0.75) | -1.33, 1.64 | 0.12 | 0.728 |  | 0.09 (0.69) | | -1.28, 1.45 | 0.10 | 0.759 |  |  |
| Group * Region | 0.10 (1.16) | -2.20, 2.40 | 0.01 | 0.934 |  | 0.16 (1.09) | | -1.99, 2.31 | 0.02 | 0.883 |  |  |
| Delay Recall |  |  |  |  |  |  | |  |  |  |  |  |
| Region | 0.73 (1.87) | -2.96, 4.43 | 2.05 | 0.154 |  | 0.06 (1.73) | | -3.36, 3.48 | 0.18 | 0.675 |  |  |
| Group * Region | 2.69 (2.90) | -3.04, 8.42 | 0.86 | 0.356 |  | 1.03 (2.74) | | -4.38, 6.43 | 0.14 | 0.708 |  |  |
| Retention |  |  |  |  |  |  | |  |  |  |  |  |
| Region | 0.26 (0.70) | -1.11, 1.63 | 1.77 | 0.186 |  | -0.27 (0.64) | | -1.54, 1.00 | 0.01 | 0.922 |  |  |
| Group * Region | 0.91 (1.08) | -1.22, 3.04 | 0.72 | 0.399 |  | 0.44 (1.02) | | -1.57, 2.44 | 0.18 | 0.668 |  |  |
| Discriminability |  |  |  |  |  |  | |  |  |  |  |  |
| Region | 0.61 (0.70) | -0.77, 1.99 | 1.29 | 0.258 |  | 0.49 (0.64) | | -0.78, 1.76 | 0.03 | 0.873 |  |  |
| Group * Region | 0.01 (1.08) | -2.13, 2.15 | 0.00 | 0.994 |  | -0.81 (1.02) | | -2.82, 1.19 | 0.64 | 0.425 |  |  |
| False Positives |  |  |  |  |  |  | |  |  |  |  |  |
| Region | 0.08 (0.71) | -1.32, 1.48 | 3.71 | 0.056 |  | 0.51 (0.66) | | -0.80, 1.82 | 0.11 | 0.746 |  |  |
| Group * Region | -2.27 (1.10) | -4.44, -0.11 | 4.28 | 0.040 | * | -1.36 (1.04) | | -3.42, 0.70 | 1.70 | 0.194 |  |  |
| Intrusions |  |  |  |  |  |  | |  |  |  |  |  |
| Region | 0.27 (0.75) | -1.22, 1.76 | 0.68 | 0.409 |  | -0.32 (0.69) | | -1.68, 1.05 | 2.15 | 0.145 |  |  |
| Group * Region | -1.50 (1.17) | -3.81, 0.81 | 1.65 | 0.200 |  | -0.97 (1.09) | | -3.13, 1.19 | 0.78 | 0.377 |  |  |

**p<0.01, *p<0.05

**Supplemental Table 5.** Univariate CVLT results: Middle temporal thickness

|  | **Left Middle temporal** | | | | | | **Right Middle temporal** | | | | | |
| --- | --- | --- | --- | --- | --- | --- | --- | --- | --- | --- | --- | --- |
| **CVLT / Effect** | **B (SE)** | **95% CI** | **F** | **p** |  | **B (SE)** | | **95% CI** | **F** | **p** |  |  |
| Total |  |  |  |  |  |  | |  |  |  |  |  |
| Region | 0.81 (0.66) | -0.49, 2.11 | 9.74 | 0.002 | ** | 0.10 (0.66) | | -1.20, 1.40 | 0.87 | 0.352 |  |  |
| Group * Region | 1.66 (1.05) | -0.42, 3.74 | 2.48 | 0.117 |  | 0.83 (1.10) | | -1.35, 3.01 | 0.57 | 0.452 |  |  |
| Learning |  |  |  |  |  |  | |  |  |  |  |  |
| Region | 0.13 (0.70) | -1.24, 1.51 | 1.14 | 0.286 |  | -0.23 (0.68) | | -1.57, 1.11 | 0.28 | 0.600 |  |  |
| Group * Region | 0.92 (1.11) | -1.27, 3.12 | 0.69 | 0.408 |  | 1.05 (1.14) | | -1.19, 3.30 | 0.86 | 0.355 |  |  |
| Delay Recall |  |  |  |  |  |  | |  |  |  |  |  |
| Region | 2.81 (1.73) | -0.60, 6.22 | 5.44 | 0.021 | * | -0.98 (1.71) | | -4.36, 2.39 | 0.00 | 0.973 |  |  |
| Group * Region | 0.82 (2.76) | -4.64, 6.27 | 0.09 | 0.768 |  | 1.87 (2.86) | | -3.77, 7.51 | 0.43 | 0.514 |  |  |
| Retention |  |  |  |  |  |  | |  |  |  |  |  |
| Region | 0.97 (0.65) | -0.31, 2.25 | 2.79 | 0.096 |  | -0.60 (0.63) | | -1.85, 0.66 | 0.55 | 0.460 |  |  |
| Group * Region | -0.21 (1.03) | -2.25, 1.83 | 0.04 | 0.837 |  | 0.41 (1.06) | | -1.69, 2.50 | 0.15 | 0.702 |  |  |
| Discriminability |  |  |  |  |  |  | |  |  |  |  |  |
| Region | 1.40 (0.64) | 0.14, 2.65 | 8.48 | 0.004 | ** | 0.09 (0.64) | | -1.17, 1.34 | 0.31 | 0.581 |  |  |
| Group * Region | 0.17 (1.02) | -1.84, 2.18 | 0.03 | 0.865 |  | 0.41 (1.06) | | -1.69, 2.51 | 0.15 | 0.699 |  |  |
| False Positives |  |  |  |  |  |  | |  |  |  |  |  |
| Region | -0.54 (0.66) | -1.85, 0.77 | 3.98 | 0.048 | * | 0.81 (0.65) | | -0.48, 2.09 | 0.01 | 0.937 |  |  |
| Group * Region | -1.04 (1.06) | -3.14, 1.05 | 0.97 | 0.327 |  | -1.52 (1.09) | | -3.68, 0.63 | 1.96 | 0.164 |  |  |
| Intrusions |  |  |  |  |  |  | |  |  |  |  |  |
| Region | -0.30 (0.70) | -1.69, 1.09 | 1.04 | 0.310 |  | 0.41 (0.69) | | -0.94, 1.77 | 0.00 | 0.964 |  |  |
| Group * Region | -0.55 (1.13) | -2.77, 1.68 | 0.24 | 0.628 |  | -0.88 (1.15) | | -3.15, 1.39 | 0.59 | 0.444 |  |  |

**p<0.01, *p<0.05

**Supplemental Table 6.** Univariate CVLT results: Precuneus thickness

|  | **Left Precuneus** | | | | | | **Right Precuneus** | | | | | |
| --- | --- | --- | --- | --- | --- | --- | --- | --- | --- | --- | --- | --- |
| **CVLT / Effect** | **B (SE)** | **95% CI** | **F** | **p** |  | **B (SE)** | | **95% CI** | **F** | **p** |  |  |
| Total |  |  |  |  |  |  | |  |  |  |  |  |
| Region | 0.56 (0.70) | -0.81, 1.94 | 3.26 | 0.073 |  | 0.33 (0.79) | | -1.23, 1.90 | 1.25 | 0.265 |  |  |
| Group * Region | 0.72 (1.02) | -1.30, 2.73 | 0.49 | 0.484 |  | 0.63 (1.15) | | -1.65, 2.90 | 0.29 | 0.588 |  |  |
| Learning |  |  |  |  |  |  | |  |  |  |  |  |
| Region | -0.04 (0.72) | -1.47, 1.38 | 0.23 | 0.632 |  | 0.30 (0.82) | | -1.32, 1.91 | 0.75 | 0.388 |  |  |
| Group * Region | 0.60 (1.06) | -1.50, 2.70 | 0.32 | 0.574 |  | 0.44 (1.19) | | -1.91, 2.79 | 0.14 | 0.713 |  |  |
| Delay Recall |  |  |  |  |  |  | |  |  |  |  |  |
| Region | -0.65 (1.81) | -4.22, 2.92 | 0.78 | 0.377 |  | 0.00 (2.06) | | -4.06, 4.07 | 0.24 | 0.627 |  |  |
| Group * Region | 3.65 (2.65) | -1.59, 8.88 | 1.89 | 0.171 |  | 1.45 (2.99) | | -4.46, 7.36 | 0.24 | 0.629 |  |  |
| Retention |  |  |  |  |  |  | |  |  |  |  |  |
| Region | -0.44 (0.67) | -1.77, 0.88 | 0.26 | 0.610 |  | -0.10 (0.77) | | -1.61, 1.41 | 0.02 | 0.897 |  |  |
| Group * Region | 1.39 (0.99) | -0.56, 3.34 | 1.99 | 0.161 |  | 0.35 (1.11) | | -1.85, 2.55 | 0.10 | 0.754 |  |  |
| Discriminability |  |  |  |  |  |  | |  |  |  |  |  |
| Region | 0.45 (0.68) | -0.88, 1.79 | 0.68 | 0.412 |  | 0.67 (0.76) | | -0.84, 2.18 | 0.12 | 0.728 |  |  |
| Group * Region | -0.09 (0.99) | -2.05, 1.87 | 0.01 | 0.929 |  | -0.95 (1.11) | | -3.14, 1.25 | 0.73 | 0.395 |  |  |
| False Positives |  |  |  |  |  |  | |  |  |  |  |  |
| Region | 0.17 (0.69) | -1.19, 1.53 | 1.30 | 0.256 |  | -0.18 (0.79) | | -1.74, 1.38 | 0.53 | 0.466 |  |  |
| Group * Region | -1.50 (1.01) | -3.50, 0.51 | 2.18 | 0.142 |  | -0.47 (1.15) | | -2.74, 1.79 | 0.17 | 0.680 |  |  |
| Intrusions |  |  |  |  |  |  | |  |  |  |  |  |
| Region | -0.17 (0.73) | -1.61, 1.28 | 0.50 | 0.482 |  | 0.03 (0.82) | | -1.60, 1.66 | 1.07 | 0.303 |  |  |
| Group * Region | -0.43 (1.07) | -2.54, 1.69 | 0.16 | 0.692 |  | -1.30 (1.20) | | -3.66, 1.07 | 1.17 | 0.280 |  |  |

**p<0.01, *p<0.05

**Supplemental Table 7.** Univariate CVLT results: Superior frontal thickness

|  | **Left Superior frontal** | | | | | | **Right Superior frontal** | | | | | |
| --- | --- | --- | --- | --- | --- | --- | --- | --- | --- | --- | --- | --- |
| **CVLT / Effect** | **B (SE)** | **95% CI** | **F** | **p** |  | **B (SE)** | | **95% CI** | **F** | **p** |  |  |
| Total |  |  |  |  |  |  | |  |  |  |  |  |
| Region | 0.26 (0.70) | -1.12, 1.64 | 0.96 | 0.330 |  | 0.46 (0.65) | | -0.83, 1.74 | 0.18 | 0.669 |  |  |
| Group * Region | 0.65 (1.20) | -1.72, 3.01 | 0.29 | 0.590 |  | -0.36 (1.29) | | -2.91, 2.20 | 0.08 | 0.783 |  |  |
| Learning |  |  |  |  |  |  | |  |  |  |  |  |
| Region | 0.15 (0.72) | -1.28, 1.58 | 0.03 | 0.866 |  | 0.25 (0.67) | | -1.08, 1.57 | 0.01 | 0.906 |  |  |
| Group * Region | -0.09 (1.24) | -2.53, 2.36 | 0.01 | 0.944 |  | -0.65 (1.33) | | -3.28, 1.98 | 0.24 | 0.628 |  |  |
| Delay Recall |  |  |  |  |  |  | |  |  |  |  |  |
| Region | 0.73 (1.81) | -2.85, 4.30 | 0.84 | 0.359 |  | 0.34 (1.69) | | -2.99, 3.67 | 0.13 | 0.719 |  |  |
| Group * Region | 1.39 (3.10) | -4.73, 7.51 | 0.20 | 0.654 |  | 0.52 (3.35) | | -6.08, 7.13 | 0.03 | 0.876 |  |  |
| Retention |  |  |  |  |  |  | |  |  |  |  |  |
| Region | 0.23 (0.67) | -1.10, 1.56 | 1.02 | 0.315 |  | 0.06 (0.62) | | -1.18, 1.29 | 0.73 | 0.396 |  |  |
| Group * Region | 0.70 (1.15) | -1.57, 2.97 | 0.37 | 0.541 |  | 0.94 (1.24) | | -1.50, 3.39 | 0.58 | 0.448 |  |  |
| Discriminability |  |  |  |  |  |  | |  |  |  |  |  |
| Region | 0.94 (0.67) | -0.39, 2.27 | 0.59 | 0.444 |  | 1.00 (0.62) | | -0.22, 2.23 | 0.02 | 0.899 |  |  |
| Group * Region | -1.00 (1.15) | -3.27, 1.27 | 0.76 | 0.386 |  | -1.85 (1.23) | | -4.28, 0.59 | 2.24 | 0.136 |  |  |
| False Positives |  |  |  |  |  |  | |  |  |  |  |  |
| Region | -0.18 (0.69) | -1.55, 1.18 | 1.47 | 0.228 |  | 0.19 (0.65) | | -1.08, 1.47 | 0.10 | 0.753 |  |  |
| Group * Region | -1.07 (1.18) | -3.41, 1.27 | 0.82 | 0.368 |  | -0.79 (1.28) | | -3.32, 1.74 | 0.38 | 0.539 |  |  |
| Intrusions |  |  |  |  |  |  | |  |  |  |  |  |
| Region | 0.38 (0.73) | -1.05, 1.82 | 0.26 | 0.614 |  | 0.15 (0.68) | | -1.18, 1.49 | 0.13 | 0.717 |  |  |
| Group * Region | -1.40 (1.24) | -3.85, 1.06 | 1.26 | 0.263 |  | -0.80 (1.35) | | -3.45, 1.86 | 0.35 | 0.555 |  |  |

**p<0.01, *p<0.05

**Supplemental Table 8.** Univariate CVLT results: Supramarginal thickness

|  | **Left Supramarginal** | | | | | | **Right Supramarginal** | | | | |  |
| --- | --- | --- | --- | --- | --- | --- | --- | --- | --- | --- | --- | --- |
| **CVLT / Effect** | **B (SE)** | **95% CI** | **F** | **p** |  | **B (SE)** | | **95% CI** | **F** | **p** |  | |
| Total |  |  |  |  |  |  | |  |  |  |  | |
| Region | 0.95 (0.63) | -0.30, 2.20 | 10.11 | 0.002 | ** | 0.85 (0.63) | | -0.39, 2.10 | 1.38 | 0.242 |  | |
| Group * Region | 1.51 (1.07) | -0.61, 3.62 | 1.98 | 0.162 |  | -0.40 (1.11) | | -2.60, 1.80 | 0.13 | 0.719 |  | |
| Learning |  |  |  |  |  |  | |  |  |  |  | |
| Region | 0.18 (0.67) | -1.14, 1.50 | 0.53 | 0.467 |  | 0.19 (0.65) | | -1.10, 1.48 | 0.29 | 0.589 |  | |
| Group * Region | 0.46 (1.13) | -1.78, 2.70 | 0.17 | 0.683 |  | 0.24 (1.15) | | -2.04, 2.51 | 0.04 | 0.837 |  | |
| Delay Recall |  |  |  |  |  |  | |  |  |  |  | |
| Region | -0.31 (1.67) | -3.61, 2.99 | 1.20 | 0.274 |  | 0.79 (1.64) | | -2.45, 4.03 | 0.12 | 0.734 |  | |
| Group * Region | 3.73 (2.84) | -1.86, 9.33 | 1.73 | 0.190 |  | -0.60 (2.89) | | -6.31, 5.11 | 0.04 | 0.837 |  | |
| Retention |  |  |  |  |  |  | |  |  |  |  | |
| Region | -0.47 (0.62) | -1.69, 0.76 | 0.29 | 0.590 |  | 0.07 (0.61) | | -1.13, 1.27 | 0.00 | 0.972 |  | |
| Group * Region | 1.51 (1.05) | -0.57, 3.59 | 2.05 | 0.154 |  | -0.18 (1.08) | | -2.30, 1.94 | 0.03 | 0.869 |  | |
| Discriminability |  |  |  |  |  |  | |  |  |  |  | |
| Region | 0.65 (0.62) | -0.58, 1.88 | 0.94 | 0.334 |  | 0.59 (0.61) | | -0.61, 1.79 | 0.08 | 0.775 |  | |
| Group * Region | -0.27 (1.06) | -2.35, 1.82 | 0.06 | 0.801 |  | -0.87 (1.07) | | -2.98, 1.25 | 0.65 | 0.420 |  | |
| False Positives |  |  |  |  |  |  | |  |  |  |  | |
| Region | 0.88 (0.64) | -0.37, 2.14 | 0.40 | 0.531 |  | 0.20 (0.63) | | -1.04, 1.45 | 0.01 | 0.934 |  | |
| Group * Region | -2.44 (1.08) | -4.57, -0.31 | 5.13 | 0.025 | * | -0.50 (1.11) | | -2.69, 1.69 | 0.20 | 0.652 |  | |
| Intrusions |  |  |  |  |  |  | |  |  |  |  | |
| Region | 0.39 (0.67) | -0.95, 1.72 | 0.09 | 0.768 |  | -0.66 (0.66) | | -1.96, 0.63 | 2.24 | 0.136 |  | |
| Group * Region | -1.11 (1.14) | -3.37, 1.15 | 0.94 | 0.335 |  | -0.40 (1.16) | | -2.69, 1.88 | 0.12 | 0.727 |  | |

**p<0.01, *p<0.05

**Supplemental Table 9.** Bayesian ROI block comparison summary by region

| **Region** | **BF10** | **BF01** | **Bulk ESS** | **Tail ESS** |
| --- | --- | --- | --- | --- |
| L Hippocampus | 2.61 | 0.38 | 6179 | 10073 |
| R Hippocampus | 3.32 | 0.31 | 5470 | 9474 |
| L Entorhinal | 0.01 | 106 | 6810 | 10660 |
| R Entorhinal | 0.00 | 609342 | 6990 | 10685 |
| L Inferior parietal | 0.00 | 569 | 5750 | 9194 |
| R Inferior parietal | 0.00 | 369 | 5975 | 10177 |
| L Middle temporal | 0.00 | 319 | 6779 | 10631 |
| R Middle temporal | 0.00 | 5924 | 7459 | 11167 |
| L Precuneus | 0.00 | 2867 | 6287 | 9959 |
| R Precuneus | 0.00 | 3083 | 5956 | 8663 |
| L Superior frontal | 0.00 | 2149 | 5637 | 9437 |
| R Superior frontal | 0.01 | 185 | 5438 | 8903 |
| L Supramarginal | 0.12 | 8.83 | 6217 | 9764 |
| R Supramarginal | 0.00 | 3000 | 5828 | 9957 |

**Supplemental Table 10.** Bayesian Group*ROI comparison summary by region

| **Region** | **BF10** | **BF01** | **Bulk ESS** | **Tail ESS** |
| --- | --- | --- | --- | --- |
| L Hippocampus | 1.68 | 0.59 | 6179 | 10073 |
| R Hippocampus | 1.26 | 0.80 | 5470 | 9474 |
| L Entorhinal | 0.02 | 43 | 6810 | 10660 |
| R Entorhinal | 0.00 | 1473 | 6990 | 10685 |
| L Inferior parietal | 0.18 | 5.69 | 5750 | 9194 |
| R Inferior parietal | 0.15 | 6.45 | 5975 | 10177 |
| L Middle temporal | 0.04 | 24 | 6779 | 10631 |
| R Middle temporal | 0.03 | 29 | 7459 | 11167 |
| L Precuneus | 0.10 | 9.71 | 6287 | 9959 |
| R Precuneus | 0.10 | 9.55 | 5956 | 8663 |
| L Superior frontal | 0.17 | 5.97 | 5637 | 9437 |
| R Superior frontal | 0.57 | 1.76 | 5438 | 8903 |
| L Supramarginal | 1.08 | 0.93 | 6217 | 9764 |
| R Supramarginal | 0.04 | 23 | 5828 | 9957 |
